# Progress in Medicine

**Published:** 1899-03-11

**Authors:** 


### Progress in Medicine.

### RHEUMATISM AND GOUT.

(*Concluded from page* 348.)

Arthur P. Luff[1] describes the method of treatment by the Greville electrothermic generator, which he has found very beneficial in gout and rheumatism. The heat is generated by passing strong currents through fine wires inside small metal cases built to enclose a single limb or joint. The temperature of the enclosed air is raised to 300 deg. or 340 deg., and the limb is kept in the hot air bath for about forty-five minutes. He suggests that possibly diffused electric currents may have something to do with the effects produced. It is clear that the increase of circulation and perspiration take a great share in the process. The temperature of the joint or limb is, of course, not raised to near that of the surrounding air owing to this protective action.

W. Taylor[2] has invented and used an apparatus for driving a current of air heated by an electric wire. This current is made to strike on the painful spot, and the effects of heated air in motion are said to be greater than that of still air of the same temperature. He found it very useful in neuralgia and local affections, and when used with volatilised substances it gave relief in various throat affections. Hot-air apparatus of various types is now largely used at the great hydropathic and bathing centres; one or two systems are to be found at Bath, and the Greville has been installed at Harrogate, where Myrtle[3] reports some remarkable results without any unpleasant effects. In place of these local baths Sydney Short[4] employs an ordinary hot-air bath for the whole body as used in most hospitals, but with an opening in the blankets to let out the hot air when saturated with moisture. With the help of this patients will bear a temperature of 200 deg., and great relief of the joint troubles and pain is obtained.

**Gout.**—Schmoll[5] thinks we are still ignorant as to what substances are retained in the body in gout, and what are the causes that produce this retention. He accepts as facts the increased richness of the blood in uric acid during gout, and indicates three sources for it: (1) The nuclein of destroyed cells; (2) the alloxan substances; (3) previous deposits of urates in gouty persons. If there is really an increased production of uric acid in gout, it is due to increased cell destruction, and this is brought about by the necrosis which accompanies the presence of crystals of uric acid in the tissues, a necrosis which is caused by retained effete matters. One argument against the retention theory of gout is the fact that a patient to whom thymus gland was given excreted an increased amount of uric acid, just as occurs in health when nucleinic acid is given. Vaughan Harley, however,[6] remarks that a great increase of leucocytes (which contain this substance) is not always accompanied by an increased excretion of uric acid, though it is frequently the case in leukemia and allied diseases, so that the relationship between nuclein and uric acid is not quite certain, especially in gout.

G. W. Balfour[7] argues that in an acute attack of gout there is a stoppage of the circulation in the capillaries around the joint, but no true inflammation, though an exudation of serum, and, indeed, of corpuscles takes place, which leaves in the tissues afterwards a deposit of urates. In support of this view he notes the sub-normal temperature of affected joints, and the occasional cures which have been obtained by massage and even active exercise. Neusser claimed that a distinctive sign of the uric acid diathesis is to be found in the presence of his basophilic granules placed on and around the nuclei of the leucocytes, and that there is an increased excretion of alloxuric bodies in the urine when these granules are present. His conclusions have been denied by Futcher and others, and recently Simon[8] has re-examined the whole subject. He finds, on the other hand, that these bodies are almost constantly present in healthy persons. In some diseases, notably in cancer, they seem to be absent, though further observations are needed here, and, finally, there appears to be no connection between the number of the granules and the amount of uric acid and xanthin bases eliminated. Thus Neusser's theory is not substantiated. The effect of gout as a factor of life assurance has been discussed by James Meikle[9] with reference to a series of 525 persons who were charged higher rates on account of their suffering from it. The mortality exceeded even that contemplated in these excess rates except after the age of 70. Thirty-three per cent. more than the expected numbers died before their time, and the author's figures went to show that the mortality requires about double the present extra premium. It was argued, however, that as his cases went back to 1863 many other things would then be included under gout and help to raise the mortality.

**Treatment.**—Luff, in his recent work on the subject, recommends colchicum, because it diminishes the production of uric acid, and therefore arrests its absorption from the kidneys and its further deposition in the tissues. Salicylates, he thinks, are actually harmful, for they increase the production of uric acid, and in the gouty state there is a great difficulty in its elimination. He opposes, too, the use of alkalies, piperazine, and lysidine as ineffective, and restricts the use of meat since its mineral salts diminish the solubility of the biurate of sodium. Vegetables have the opposite effect, and delay the conversion of the quadriurate into the biurate. Among the best of these for gouty patients are spinach, Brussels sprouts, savoys, turnips, celery, winter cabbage, and French beans. The injurious action of some wines is not due to their acidity, but to some effect they produce on the liver. Indeed there is no general acidity of the blood or of the system in gout, and the administration of alkalies does not render the deposits more soluble.

Levison[10] gives chiefly colchicum and quantities of boiled water and mild diuretics, and praises very highly the use of electrical baths for the injured joints and the tophi. He has succeeded in restoring movement, lessening deformity, and getting rid of pain in a large number of cases of chronic gouty joints by kataphoresis, or the introduction through the skin of lithium salts directly over the damaged joint. The patient places the injured hand in a solution of lithium, and the other hand, or a foot, in a solution of common salt. The positive pole of a battery is placed in the lithium, but away from the hand, and the negative pole in the other solution. A large amount of lithium is driven into the diseased tissues, and acts on them, later on appearing in part in the urine. His successes are so numerous, and the results so lasting, that the method deserves a further trial. However, he warns us that we must have made a correct diagnosis, for similar good results do not follow with joints damaged from other causes.

**Purpura and Hæmophilia.**—A recent writer, in speaking of the difficulty of restraining bleeding after tooth extraction, mentions that he succeeded in stopping it in a hæmophiliac patient by spraying the gum and the cavity with ethylene chloride, so as to freeze the blood at once as it issued. W. H. Brown[11] records a case where death was imminent from the hæmorrhage from a wound, and the patient's six brothers had all died from the same cause. Large [illegible]ntities of oxygen were given by inhalation, and the bleeding quickly ceased. The remedy was continued for a week, and the patient recovered completely. Leon Perrin[12] regards all purpuras in children as signs of a toxæmia, the rheumatic forms differing only in being produced by another toxin from the common septic ones. Whether the germs enter through the tonsils, the skin, or the intestines, their action on the blood is the same. Certain drugs, indeed, have a similar effect. Purpura he finds, resembles the eruptive fevers in attacking children more often than adults, the most common age being from four to ten. F. A. Thompson[13] reports a case of Henoch's purpura in a girl aged eleven. She was attacked with cramps and vomiting. Much blood was passed by the bowel and vomited; the temperature was normal. The elbows, knees, thighs, and right wrist were swollen and covered with petechiæ. The child soon recovered, and has had two or three subsequent attacks. A similar case is reported by E. R. Dawson[14] in a girl of two years and eleven months. Slight epistaxis was followed by hæmaturia, petechiæ, much vomiting of blood, and abdominal pain. Ergotine injections seemed to give some relief, and after a month's illness complete recovery followed.

[1] Practitioner, Feb. [2] Lancet, Nov. 26. [3] Brit. Med. Jour., Dec 10. [4] Brit. Med. Jour., Nov. 26. [5] Brit. Med. Jour., Nov. 26. [6] Philadelphia Med. Jour., Sept. [7] Lancet, Aug. 10. [8] Amer. Jour. Med. Sci., Feb. [9] Brit. Med. Jour., Sept. 17. [10] Practitioner, Sept. [11] Lancet, Dec. 3. [12] Lancet, Dec. 10. [13] New York Med. Jour., Nov. 26. [14] Lancet, Jan. 21.

